# Statistics of multiscale fragmentation in the Primorsky fault zone

**DOI:** 10.1038/s41598-026-47316-w

**Published:** 2026-04-13

**Authors:** Alexey Ostapchuk, Vladislav Chinkin, Antonina Grigorieva, Dmitry Pavlov

**Affiliations:** 1 Sadovsky Institute for Dynamics of Geospheres, Russian Academy of Sciences, Moscow, 119334 Russia; 2 Institute of Geology of Ore Deposits, Petrography, Mineralogy and Geochemistry, Russian Academy of Sciences, Moscow, 119017 Russia

**Keywords:** Tectonic fault, Brittle deformation, Lognormal statistics, Scaling, image segmentation, Kolmogorov fragmentation theory, Natural hazards, Solid Earth sciences

## Abstract

Tectonic stresses cause rock deformation and disintegration. We examined the fragmentation statistics of brittle rocks composing the damage zone of the Primorsky Fault of the Baikal Rift Zone at scales ranging from microns to kilometers. The fault rocks analyzed include different lithologies and shear-strain magnitudes. We use the convolutional neural network algorithm to automate the mapping of fractures in images and faults in topographic data and statistically test for presence of power, lognormal or Weibull laws. Fault-rock fragmentation obeys lognormal statistics at scales from 10^− 6^m to 10^4^m, and the shape parameter (σ) is preserved and varies in the range 1.4–2.0. We demonstrate that summarizing the truncated data may lead to compilation artifact and incorrect conclusions about the power law behavior. We proposed a statistical fragmentation model to fit to experimental logarithmically distributed data. At all scales the rate of destruction depends on the fragment size as a power law. Findings should be incorporated in models estimating fault geometry characteristics and evolution of earthquake source.

## Introduction

Deformation processes in the brittle crust involve scales from crystals (10^− 8^ m) to lithospheric plates (10^7^ m). Microcracking and fracturing may develop as a part of a single deformation event, in which case they are likely to have interrelated kinematics and mechanics, or during a sequence of events under different boundary conditions^1–3^. Tectonic faults are the areas of the most active deformation processes.

The major crustal faults are generally tabular structures and exhibit complex internal architectures. The structure of a tectonic fault rock depends on the depth, properties of the host rock, tectonic conditions, cumulative deformation, etc.^4,7–12^. The evolution of the fault fabric is accompanied by changes in fault slip and hydrological behavior^13,14^. A mature fault has two structural features, a damage zone and a fault core. The damage zone, in general, is defined by the frequency distribution of damage structures, such as cracks, fractures and deformation bands, which commonly decrease with distance from the fault core^4^. The widths of damage zones are typically positively correlated with displacement. The fault core is the result of highly localized strain and intense shearing that accommodates the majority of the displacement within the fault zone^5^. Depending on the rheological properties of the host rocks, fault core may contain a mixture of materials with varying competence deforming by different deformation modes or several principal slip zones with recurring slip surfaces and fault rocks such as gouges, cataclasites, and breccias^6^. The fault zone involves seismogenic slip along the main fault surface and deformation within a volume around that surface, both of which accumulate over time^6^. The higher the values of accommodated displacement are, the greater the degree of rock fragmentation fineness. Within the fault zones, there are areas with different accumulated strains and different degrees of fragmentation. Highly intense seismic slip localized in narrow principal slip zone causes zircon fragmentation^15^.

Many studies have been devoted to measuring and analyzing the fragment size distributions of fault rocks^16–19^. The basic mechanism of the theory of fragmentation was described by A.N. Kolmogorov^20^. In 1947, B. Epstein explicitly formulated the conditions under which fragments are distributed according to the lognormal law at large times^21^:

1. The probability of destruction of any particle at any stage of the process is independent of its size, availability of other particles and number of stages of crushing.
2. The distribution of particles after a single crush over relative sizes is independent of the size of the virgin particle.

As a part of the fractality of geomedium, it was suggested that the fragmentation is a self-similar process, causing a power law distribution of particles over sizes^22,23^. Statistical verification shows that the power statistics are less accurate and lose to lognormal statistics or the generalized gamma distribution^24,25^. In many studies, particles smaller than a size threshold are excluded from the analysis; so the results basing on truncated datasets compilations from different sources are discrepant^25^.

The development of digital observation technologies (electron microscopy, digital photography, cosmic geodesy, aerial photography, etc.) delivers a large amount of factual material. The automatic detection of structures is widely used in analysis. Deep learning algorithms are used to detect mineral grains^26,27^, determine the segmentation of thin section^28,29^ and fault scarps^30,31^ and analyse topographic data^32,33^. The availability of effective algorithms for segmenting images allows the analysis of a large amount of observation data within a uniform framework.

In this work, we investigate the regularity of fragmentation of the rock composing the damage zone and the core of the Primorsky Fault of the Baikal Rift Zone. Rock datasets include fragmented zircon grains collected in the principal slip zone, thin sections collected along the fault strike in the fault core, rock outcrops at different distances from the fault core and a digital elevation map of the Baikal region. All the data under analysis concern one and the same mature tectonic fault and cover the scales 10^− 6^–10^4^ m. An automatic segmentation of images and a statistical analysis of fragment size have been performed. The fragmentation was mapped in two dimensions. The analysis included lognormal, power and exponential statistics, which correspond to the common two-parameter models of rock fragmentation^34,35^. A kinetic model of fragmentation was constructed based on statistical fragmentation theory; the model describes the statistical features of the experimental data.

## Results

### Geological setting and sample location

The object of investigation is the main collisional suture between the Siberian Craton and the Olkhon Terrane^36^. The Primorsky Fault formed inside this collisional suture during the genesis of the Cenozoic rift^37^. During the neotectonic stage, the Primorsky fault had formed as a normal fault with a left-lateral oblique shear component. Tectonic deformation was accompanied by fluid penetration, which promoted the extraction of carbon matter within the fault core^38^. Metamorphic and magmatic complexes exhumed from seismogenic depths of 15–25 km are now accessible for field exploration^39,40^. Segments with different slip behaviors have been revealed along the strike of the Primorsky Fault^41^.

**Fig. 1:**
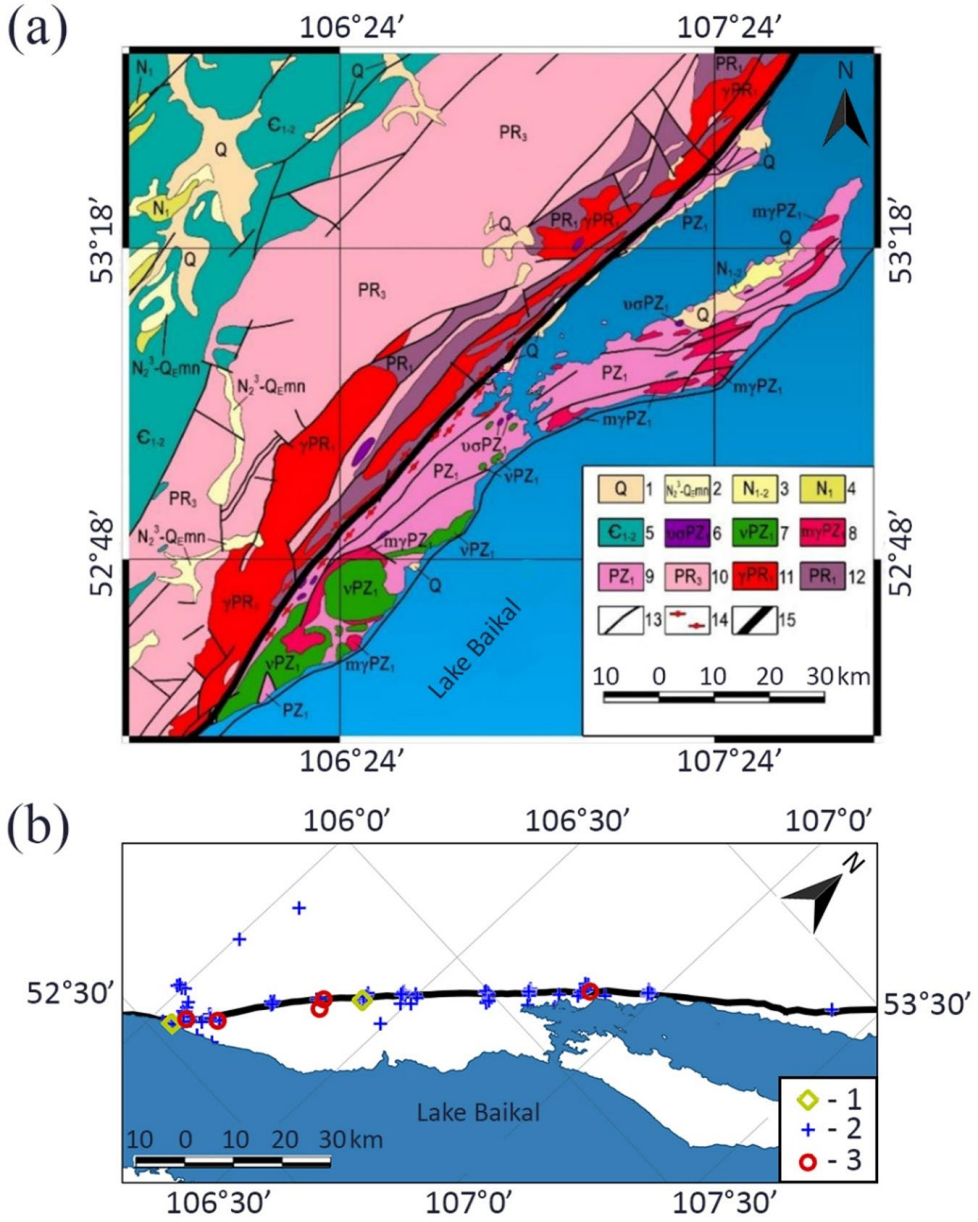
Geological map of the Primorsky Fault and sample collection sites. (**a**) Geological map^37,42^: 1 – beach lacustrine deposits of Lake Baikal and alluvium of river valley terraces, 2 – Manzurka Fm. Alluvium, 3 – Golumets Fm. Alluvium, 4 – Bayanday Fm. Alluvium, 5 – Lower and Middle Cambrian sediments on the Siberian craton, 6 – Atarkhan peridotite–gabbro complex, 7 – Ozersk gabbro–diorite complex, 8 – Olkhon migmatite–granite complex, 9 – Early Paleozoic metamorphic complexes of the Olkhon terrane, 10 – Upper Proterozoic sediments (Baikal Group), 11 – Primorsky granite complex, 12 – Early Proterozoic craton basement, 13 – main faults, 14 – blastomylonite from the marginal suture of the Siberian craton; 15 – Primorsky Fault. (**b**) Map of sample collection: 1 – zircon grain, 2 – thin section, 3 – rock outcrop.

Field exploration data include information about a segment of the Primorsky Fault that is 160 km long (Fig. 1). The minerals, rocks and fragments of outcrops, which are characterized mainly by brittle deformation that occurred under tectonic stresses, were considered. Oriented rock samples were collected along a 160-km segment of the fault strike. Thin sections were made of the samples, and zircons were collected after crushing the samples. Rock outcrops were frontal photographed and orientation of bedding was determined during field observations. Information about the factual material used is presented in Table 1.

**Table 1:** Description of sample data.

Object	Zircon	Rock thin section	Rock outcrop	Digital elevation model
Image of data				
Image resolution	2.55–5.0 px/um	4 px/um	69.5–243.3 px/m	120 m/px
Amount of data	10 damaged grains of zircon of the total collection of 66 grains.	234 fragmented thin sections No segmentation was performed.	5 rock outcrops of a characteristic size of about 3 × 3м^2^	5 sections with a map of lineaments. No detection of lineaments was performed
Source of data	Photos of zircons available at Mendeley Data^43^ are used as the initial data.	Data on statistics of rock fragmentation was borrowed from the Ref.^44^	Photos of outcrops available at Mendeley Data^45^ are used as the initial data.	Lineaments of DEM are used as the initial data. The data is presented in Ref.^46^.

Zircons are considered markers of fragmentation at the mineral level. Zircon has a high density and hardness, which is determined by its crystallographic properties^47^. It is stable in metamorphic processes^48^. Therefore, zircon preserves the mechanical deformation that it had undergone at different stages of tectonic activity and prevents mechanical crushing.

**Fig. 2:**
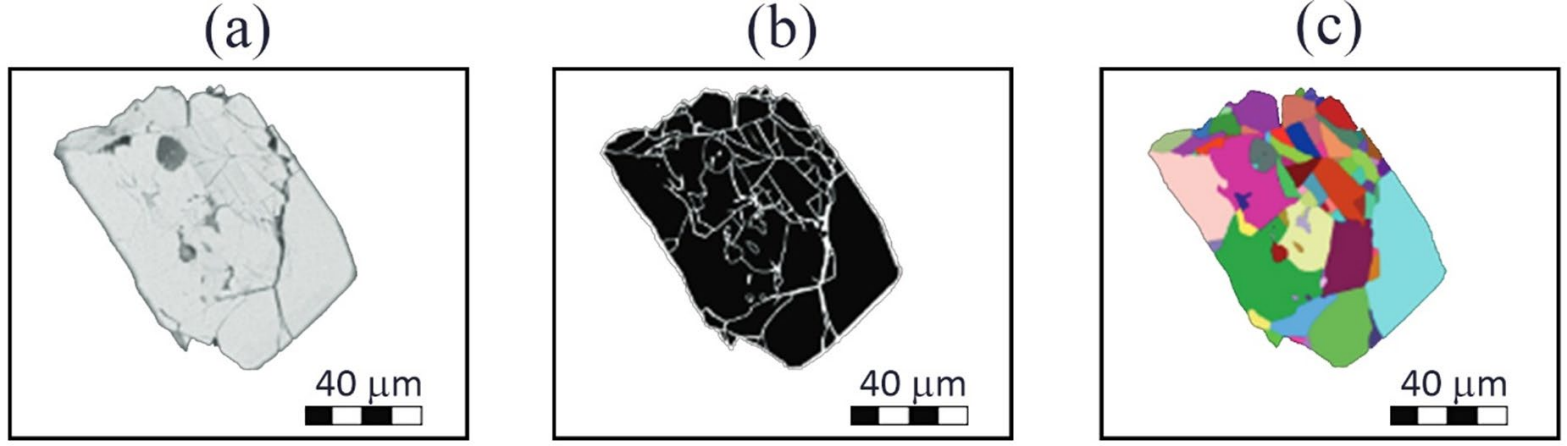
Fragmentation of zircon grain. (**a**) initial image in the regime of reflected electrons; (**b**) the detected boundaries of segments (cracks); (**c**) mapping fragmentation of zircon grain.

Images of the grains, obtained at a scanning electron microscope, clearly show cracks in cross-sections (Fig. 2a). The image resolution allowed us to detect all cracks. The cracks are randomly distributed throughout the grains and are not related to cleavage direction. Zircon grains were released from the rock during jaw crushing, and after crushing they retained their shape. A comparison of zircon grains in thin sections and individualized zircon grains, obtained after crushing, showed that the level of fracturing in the grains is identical. For the analysis zircons were selected that were fragmented in more than 20 pieces . This threshold allowed us to focus on strongly fragmented grains while preserving the maximum possible number of samples.

Mechanical destruction in rocks was determined in thin sections. Brittle deformation is characteristic of rocks such as granite, granite gneiss and plagiogranite, where quartz and feldspar prevail. The most brittle minerals are crushed first under stress, in this case it is quartz. Under high deformations it is easily crushed to the finest bits of irregular shape that form the matrix. Feldspars have a more complex frame structure and stronger crystalline bonds. Under similar tectonic impacts on rocks, quartz can be crushed into tiny xenomorphic particles, while feldspar can remain in the newly formed rocks as larger grains, porphiroblasts. The dataset of the fragmentation of thin sections is presented in the work^44^.

Most of investigated outcrops were formed of metamorphic rocks. In these rocks, the initial destruction processes can hardly be traced because of recrystallization and the emergence of new minerals. Outcrops retrieved brittle deformations obtained under mechanical effects. The rocks were granites and granite gneisses. Resolution of outcrop photography makes it possible to identify cracks on a centimeter scale.

Lineament analysis data from a digital elevation map (DEM) were used to analyse the large-scale faultedness of the Earth’s crust. The map of lineament network reflects the aggregate of previous stages of tectonic evolution of the Western Baikalia^46^. The map was divided into regions whose boundaries were large tectonic faults. The high spatial resolution of the initial digital elevation map has allowed to detect a large number of lineaments of different lengths and orientations. The azimuths of the lineaments’ strikes at all scales are consistent with the strikes of the corresponding faults in the local area^46^. Although the lineaments represent now-active faults only to some extent, the scheme reflects the general zone–block structure of the crust in the region^49^.

**Fig. 3:**
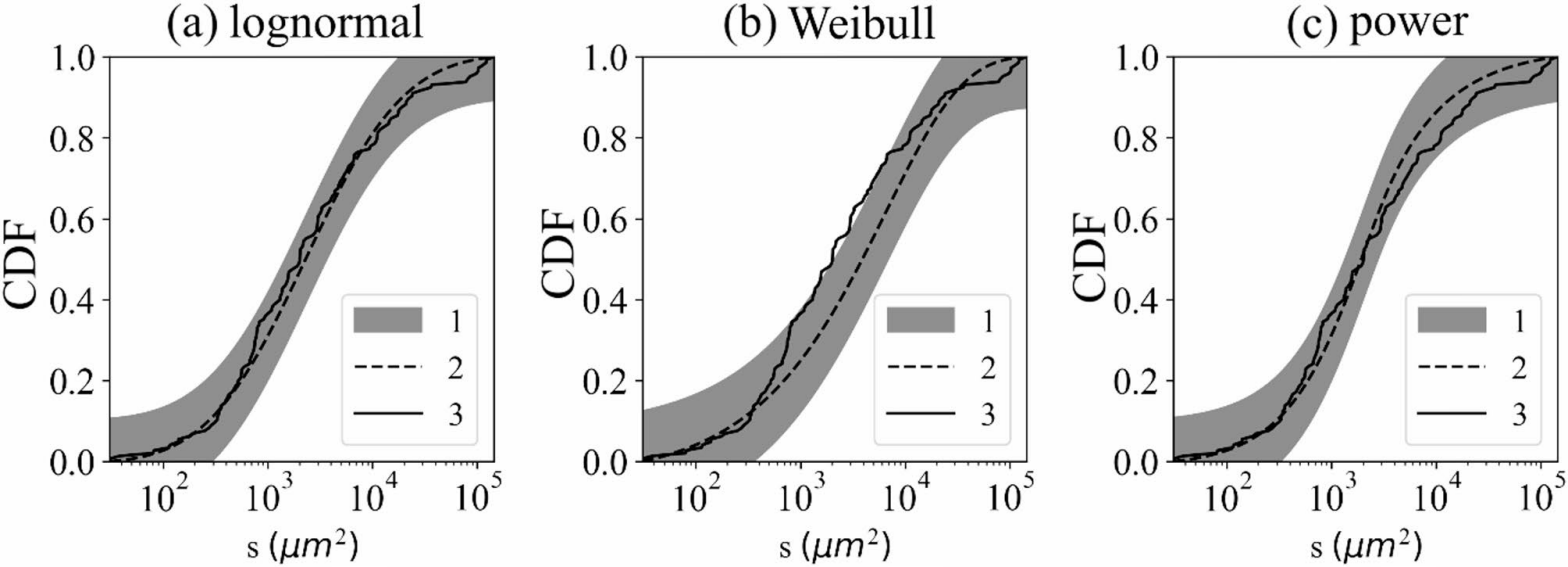
Function of probability of sizes of fragments obtained in different models of destruction (by the example of zircons). (**a**) lognormal distribution; (**b**) Weibull distribution; (**c**) power distribution. 1 – confidence interval (significance level of α = 0.05), 2 – cumulative distribution function (CDF) of fragments fitted via maximum likelihood estimation, 3 – empirical cumulative distribution function of fragments.

The precise mode of fragmentation exerts sensitive control on the resulting probability distribution for fragment size. In the frames of the statistical analysis, we used lognormal, power and Weibull distributions. These distributions reflect the main common models of rock crushing. To assess the correspondence of empirical data to the theoretical models, we applied the Kolmogorov–Smirnov (K-S) goodness-of-fit test. Fragmentation is expected to exhibit short-range spatial correlations and their overall influence decreases as the sample size increases, so that the samples may be treated as weakly dependent. Under these conditions, K-S test leads to an increased rejection rate and the corresponding p-values remain informative. Figure 3 presents zircon fragmentation statistics and the results of testing the null hypotheses, namely, that the fragment sample distributions follow the three selected theoretical models. Table 2 summarizes the results of the K-S goodness-of-fit test for three candidate distributions (9)-(11). Among the three null hypotheses tested, the lognormal distribution hypothesis was the least likely to be rejected by the K-S test at the significance level of α = 0.05 over all the data types. There are also separate objects that correspond to two models concurrently or to none of the presented distributions.

**Table 2:** Kolmogorov–Smirnov goodness-of-fit test results. The number and percentage of cases where the null hypothesis for each distribution was not rejected at the significance level of α = 0.05.

Type	Total number	Lognormal distribution	Power distribution	Weibull distribution
Zircon	10	7 (70%)	4 (40%)	4 (40%)
Thin section	234	104 (44%)	5 (2%)	1 (0.4%)
Rock outcrop	5	4 (80%)	0 (0%)	1 (20%)
DEM	5	5 (100%)	3 (60%)	0 (0%)

### Compiling the data

**Fig. 4:**
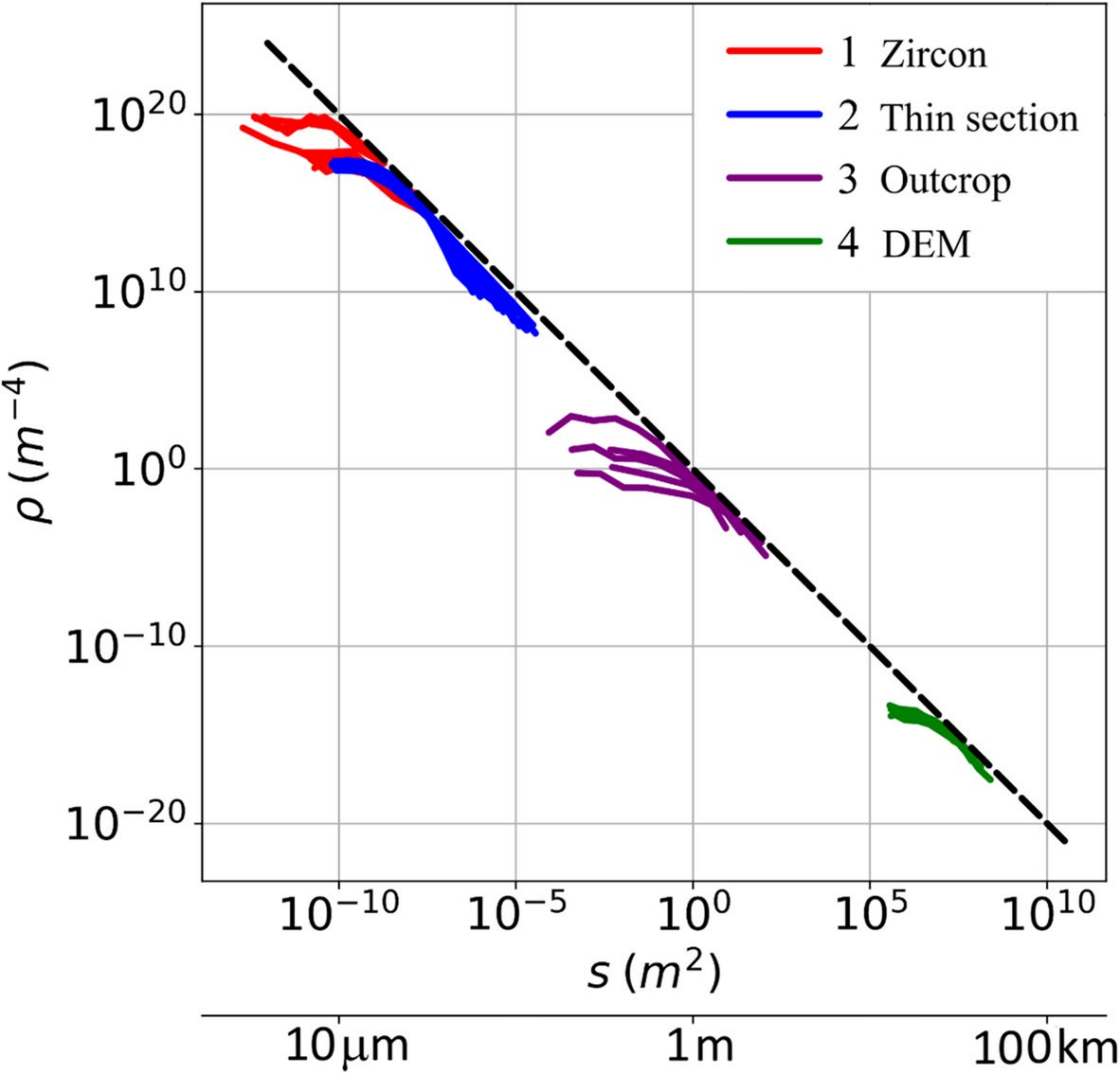
Compiling experimental data of different scales. The Y-axis shows the probability density (ρ) of fragment size scaled by the image size. 1 – zircon grain, 2 – thin section, 3 – rock outcrop, 4 – DEM. Dashed line corresponds to the dependence $\:\rho\:\left(s\right)\sim{s}^{-2}$.

All the data are associated with the long-term evolution of the Primorsky Fault. This allows us to link all the data to reveal specifics of tectonic fragmentation of the crust at different scales. Figure 4 presents distributions of the fragments over size per area unit. Despite some gaps, the data spread over 12 orders of magnitude.

It is seen in Table 2 that at all the scales the most probable hypothesis is that the fragmentation of rock massif obeys the lognormal distribution law. All the distributions show close values of shape parameters (σ). Parameter σ at the significance level of 0.05 vary in the limits from 1.4 to 2.0, and its most probable value for zircons is σ = 1.7 (thin sections – 1.6, outcrops – 1.8, DEM – 1.5).

The proximity of values of σ for different distributions can impel to plot a unified ultimate envelope, which is described by a power law (dashed line in Fig. 4). This shows that if one considers only truncated data, he can make a false conclusion that a power distribution is applicable to all the scales, and consequently, the process is self-similar^25^. However, the power law is a compilation artifact and statistically does not describe the full amount of observation data.

## Discussion

The geometrical and structural properties of brittle fault rocks are very difficult to characterize in the subsurface. Field exploration is often performed at passive faults, whose structure is a result of regional tectonics, so distinguishing primary deep processes from new superimposed processes is difficult. The Primorsky Fault experienced many stages of activation and undergone different hydrological, geochemical and mechanical impacts^37,38,40^. The fault rocks include a wide range of lithologies, and neighboring rocks may be characterized by different shear–strain magnitudes.

The patterns and degree of fragmentation of a rock under tectonic stress are associated with the formation of its fault-block structure^50–52^. Initially, fracturing in the crust is unevenly distributed and individual cracks emerge. Damage zones are the products of pre-faulting strain, fault propagation, displacement, and linkage processes taking place during the growth of the fault zone^50^. Riedel shears, P- and Y-cracks form during further evolution. Reorientation and consolidation of cracks in a local area is observed, which leads to greater fragmentation of the massif in linearly elongated areas. Subsequently, the deformation is localized along the fault core, within which the rock has maximum fragmentation^53^. Progressive fragmentation, localized in a narrower zone, forms a fault-block structure of a lower hierarchical level. Figure 4 compiles data on the evolution of fault-block structure of the Western Baikalia and the Primorsky fault. Within one and the same region, a higher fragmentation of a local area is produced by a higher accumulated deformation. As the amplitude of tectonic deformation grows, areas with high concentration of fracturing emerge, and the degree of fragmentation increases.

Using the K-S test, it was found that the datasets are largely consistent with lognormal statistics over all scales, while the alternatives are highly likely to be rejected. And log-normal fits to fragment-size distributions show that the shape parameter preserve at all scales and hierarchical levels. Revealing the fragmentation law is of critical significance to understand the evolution and properties of tectonic faults. For example, the lognormal distribution assumes, that the proportion of ultra-fine particles is very small. The low proportion of ultra-fine particles has an essential effect on the permeability of layers that had gone through noticeable deformations^54–56^. Also, the low proportion of ultra-fine particles means that the relative energy, that goes to destruction in an earthquake, is lower than it would have been if the distribution was a power one^57,58^. Choosing the power law distribution was justified by using the model of constrained comminution for the cataclastic deformation^23^. However, the lack of reliable evidence of the power distribution of particles over sizes disputes the applicability of the model. That is, partially or completely, suggestions made in the model are incorrect.

### Kinematic model of fragmentation

The three major competing views on the essential geometrical, mechanical, and mathematical natures of faults are continuum-Euclidean, granular, and fractal^1,21^. The fragmentation of the geomedium considered above suggests that there are collections of planar or tabular Euclidean zones in a continuum solid in the fault zones. The long-term deformation of a brittle solid governed by a strain weakening rheology will still be dominated by Euclidean structures of sizes comparable to those of the overall medium dimensions, surrounded by a more-or-less continuum matrix that contains a variety of fewer structures^1^. From the point of view of kinetics, the process of fragmentation can be presented in the frames of statistical fragmentation theory as follows^34,59^

1 $$\:\frac{\partial\:}{\partial\:t}c(x,t)=-R\left(x\right)c(x,t)+{\int\:}_{x}^{\infty\:}R\left(y\right)K(x,y)c(y,t)dy$$

where *R*(*x*) is the rate at which particles of size x break, and *K* (*x*, *y*) is the fragmentation distribution of particles of size *x* from the fragmentation of a rock of size *y*. Using different cores *K* (*x*, *y*) and probability rates *R*(*x*) allows the reproduction of major empirical descriptors to describe various natural processes^34,60,61^.

Let us consider the evolution of fragmentation from a monodisperse condition, which can be written as:

2 $$\:c\left(x,t\right)={N}_{0}\delta\:\left(x-{x}_{0}\right).$$

Here, we use the core of the equation in the form of Turcotte’s core:

3 $$\:K\left(x,y\right)=2\delta\:\left(x-\frac{1}{2}y\right),$$

which corresponds to the division of fragments in half. In this case the solution is as follows:

4 $$\:c(x,y)={\sum\:}_{n=0}^{\infty\:}{c}_{n}\left(t\right)\delta\:\left(x-{2}^{-n}{x}_{n}\right)$$

where ${c_n}(t)$ is the number of particles that are $\:{2}^{-n}$ times smaller than the initial size x_0_. If we consider Kolmogorov’s model, the rate of destruction suggests the following view^21^:

5 $$\:R\left(x\right)=p$$

The solution of Eq. (1) under conditions (3) and (5) and initial monodisperse condition (5) is as follows^62^:

6 $$\:{c}_{n}\left(t\right)={N}_{0}{2}^{n}\frac{(pt{)}^{n}}{n!}{exp}(-pt)$$

**Fig. 5:**
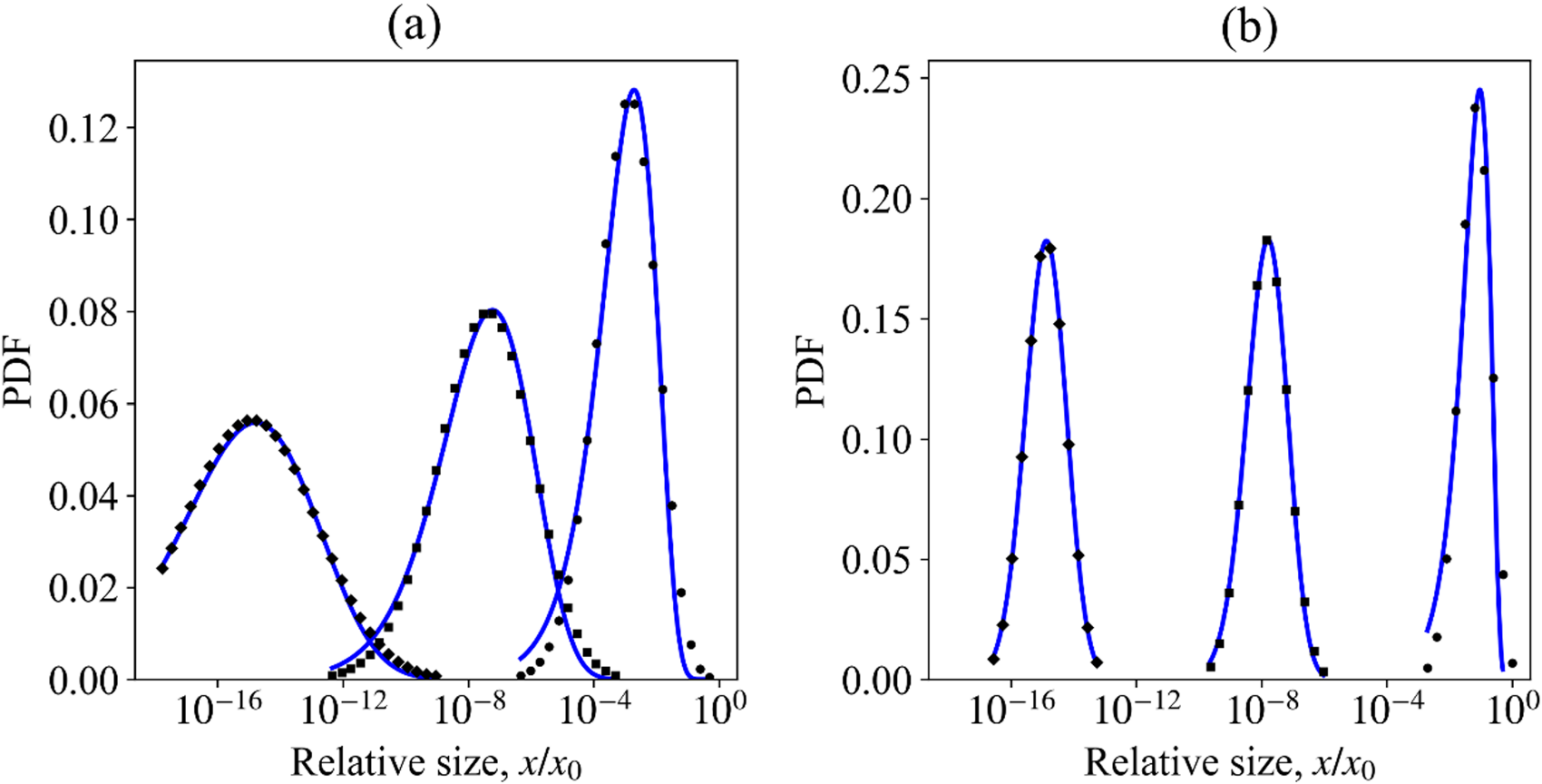
Evolution of rock fragmentation statistics in the frames of the statistical fragmentation model. The initial condition of the model – all the fragments are of the same size *x*_0_. (**a**) probability rate of destruction is $\:R\left(x\right)=const$ (Kolmogorov model); (**b**) probability rate of destruction is $\:R\left(x\right)\sim{x}^{0.9}$.

Kolmogorov model of fragmentation always leads in the limit of large times to the lognormal law of distribution; however, the value of σ increases as the mean size µ decreases (Fig. 5а). *The increase in σ contradicts the observed data.*

As an alternative, we consider the model of fragmentation, in which the probability of destruction of large particles is greater than that of destruction of small particles. Indeed, large particles have numerous defects; consequently, their mechanical strength is lower^63,64^. Let us suggest that the probability rate *R*(x) obeys a power law:

7 $$\:R\left(x\right)=p\cdot{x}^{\gamma\:}$$

The solution of Eq. (1) under conditions (3) and ( can be written as follows^65^:

8 $$\:{c}_{n}\left(t\right)={N}_{0}{2}^{n}{\sum\:}_{k=0}^{n}\frac{{2}^{\frac{\gamma\:k\left(k-1\right)}{2}}{exp}(-{2}^{\gamma\:\left(k-n\right)}pt)}{{\prod\:}_{j=\left[0;n\right]\backslash\:\left\{k\right\}}\left({2}^{\gamma\:j}-{2}^{\gamma\:k}\right)},$$

The numerical solution for function (8) is presented in Fig. 5b. Unlike Kolmogorov’s model, the shape of the curves is preserved irrespective of the number of fragmentations, i.e., irrespective of the mean size of the fragments. The lognormal law describes the fragmentation statistics well (Fig. 5). With a decrease in the mean size of the fragments, the root mean square error (RMSE) decreases; RMSE is 8⋅10^− 4^ with a decrease in the mean size by a factor of 10^8^. The shape parameter of the lognormal distribution increases with decreasing exponent γ (relation 7).

Within one and the same region, a higher degree of fragmentation in a local area, which is expressed in decreasing mean size of fragments, is produced by an accumulated deformation of a higher magnitude, the tectonic mechanism of fracturing being retained. The proposed statistical fragmentation model, despite its simplicity, adequately describes the experimental data. The model should be considered only as a basic principle of evolution of a fault-rock structure. In future, the model should incorporate mineralogy-driven variations of particle strength and fracture probability^66,67^ and consider the effects of fluid-rock interaction^12,68^ and other factors, which will cause deviations from the basic statistics.

## Methods

### Segmentation of images

Manual mapping of fractures and faults is the most accurate processing method, but it has extremely high time costs. We use an image segmentation algorithm for zircons and outcrops that contains 4 main stages:

- *Estimating object boundaries*. The Richer Convolutional Features (RCF) algorithm was used to estimate boundaries^69^. RCF bases on the VGG16 neural network and modifies this structure by removing the fully connected layers and adding side outputs for each convolutional layer, grouped into five stages. The VGG16 has 13 layers, 3 of which are fully connected and linked in series. The RCF algorithm was pre-trained on the BSDS500 dataset, with weights provided by the authors^70^. After that, fine-tuning was performed on the following datasets: Orthomosaic of Granite Dells and Valley of Fire Fault Mapping^71^, Orthomosaic of Loviisa shoreline outcrops^72^, Cross-polarized petrographic image dataset^73^. The application of RCFs results in matrices whose dimensionality coincides with the dimensionality of the initial images, and the values in the cells vary between 0 and 1. Script of algorithm is available at https://github.com/yun-liu/RCF.
- *Thresholding Edge Probability Maps.* The matrices are interpreted as maps of probabilities of boundaries. For the boundaries to be detected, the probability maps are binarized via the Otsu method^74^.
- *Detecting closed areas.* In detecting closed areas, all areas inside the area surrounded by the cells of the boundary were considered separate objects. Spaghetti labelling was used for detection^75^.
- *Expanding closed areas.* All the objects were expanded until they contacted each other. The width of the boundary was minimized to one pixel. The expansion was performed via the watershed algorithm^76^.

After automatic segmentation, visual control of the segmentation quality was performed. For the DEM scale, the initial data are lineaments, represented in Ref.^46^. For the lineaments of the DEM, we used only the 2nd and 3rd stages. In addition, the results of segmentation of thin sections were borrowed from our previous work^44^ and are used in this work to link fragmentation at different scales.

### Statistical models

The lognormal distribution was used in the following form of a probability density function (PDF)^25^:

9 $$\:f\left(s|\mu\:,\sigma\:\right)=\frac{1}{s\sigma\:\sqrt{2\pi\:}}exp\left(\frac{-{\left(ln\left(s\right)-\mu\:\right)}^{2}}{2{\sigma\:}^{2}}\right)$$

The power distribution was used in a modification of the PDF suggested by A. Clauset et al.^77^, which provides convergence of the normalization near zero:

10 $$\:f\left(s|\lambda\:,\alpha\:\right)=\left\{\begin{array}{cc}C{\left(\frac{s}{\lambda\:}\right)}^{\alpha\:}&\:,s\geqslant\lambda\:\\\:Cexp\left[-\alpha\:\left(s/\lambda\:-1\right)\right]&\:,s<\lambda\:\end{array}\right.$$

where λ is the boundary value at which the distribution transitions to a power distribution and where C is the normalization constant. This modification allows the integration of distribution (10) from zero. The PDF of the Weibull distribution was specified as follows^25,78^:

11 $$\:f\left(s|\lambda\:,\alpha\:\right)=\frac{\alpha\:}{\lambda\:}{\left(\frac{s}{\lambda\:}\right)}^{\alpha\:-1}exp\left[-{\left(\frac{s}{\lambda\:}\right)}^{\alpha\:}\right]$$

The parameters of the distribution were assessed via maximum likelihood estimation (MLE). To examine the composite hypothesis that the sample (ensemble of fragments) follows one of the selected theoretical distribution functions, the Kolmogorov–Smirnov (K-S) goodness-of-fit test was employed^79^. Python scripts of K-S test are available at GitHub via link https://github.com/IDGRockFragmentationAnaliz/pyrockstats. The significance level of α = 0.05 was employed to evaluate consistency with the null hypotheses.

### Kolmogorov-Smirnov goodness of fit test

The verification of hypothesis that the sample of segments $X=\{ {x_i}\} _{{i=1}}^{n}$ is described by the chosen theoretical model, that is described by cumulative distribution function ${F_t}(x|\theta )$ (CDF) or probability density function ${f_t}(x|\theta )$ (PDF), is based on Kolmogorov – Smirnov (K-S) goodness-of-fit test.

The method of maximum likelihood estimation (MLE) is used to estimate the parameters of model. The likelihood function of the theoretical function *f*_*t*_(*x*) and sample *X* are used:

12 $$L[{f_t},X](\theta )=\frac{1}{N}\sum\limits_{{i=1}}^{n} {\ln } {f_t}({x_i}|\theta )$$

The estimation is performed by minimizing the functional with account for the minimal *x*_*min*_ and the maximal *x*_*max*_ limitations:

13 $$L[{f_t},X](\theta )+\ln [F({x_{max}}) - F({x_{min}})] \to \hbox{min} .$$

Minimizing the functional was performed by the Nelder–Mead method with the initial point, that was calculated via analytical equations, which do not account for limitations.

Verification of a hypothesis that the sample of segments $X=\{ {x_i}\} _{{i=1}}^{n}$ is described by the chosen theoretical model was performed in several stages:

1. Estimation of parameters of the theoretical function $\hat {\theta }$ is performed via the MLE.
2. The K-S statistics is calculated for the initial sample:

14 $${D_0}=\mathop {\hbox{max} }\limits_{x} |{F_0}(x) - {F_t}(x|\hat {\theta })|$$

where ${F_0}(x)$ is the empirical cumulative distribution function (ECDF) for the sample of segments.

3. Constructing the estimation of distribution of K-S statistics is performed for different realizations of the distribution. Methods of this estimation are based on parametric bootstrap method of estimating the distribution of K-S statistics.
4. Adoption of the hypothesis at the level of significance of $\alpha$ is performed, if this statistics is less than (1 – *α*) of the quantile of distribution of K-S statistics.

15 $${D_0}<{q_{1 - \alpha }}(\{ {D_k}\} _{{k=1}}^{K}).$$

### Bootstrap method of estimating the distribution of K-S statistics

In cases, if the systematic error of segmentation is zero or minimal, we can perform an estimation of distributions of K-S statistics. This estimation is performed in several stages:

1. The bootstrap ensemble *X*_*j*_ of the same length as the one of the initial sample *X* is generated by randomly extracting values from the initial sample *X*.
2. Estimation of parameters for the theoretical cumulative function ${F_t}$ is calculated via MLE $\:{\widehat{\theta\:}}_{j}$ for each element of the ensemble *X*_*j*_.
3. A new pseudo-ensemble of random samples $\:{\widehat{X}}_{j}$ of the same length as $\:{X}_{j}$ is generated from the estimation of the theoretical cumulative distribution $\:{F}_{t}\left(x\right|{\widehat{\theta\:}}_{j})$. This guarantees that the pseudo-ensemble $\:{\widehat{X}}_{j}$ is distributed according to the theoretical distribution law ${F_t}$.
4. A corresponding EDCF $\:{F}_{j}\left(x\right)$ is constructed for each pseudo-ensemble $\:{\widehat{X}}_{j}$.
5. The sample of K-S statistics can be written as follows:

16 $${D}_{j}=\mathop{\hbox{max} }\limits_{x} |{F_t}(x|{\widehat{\theta\:}}_{j}) - \hat{F_j}(x)|$$.

## Data Availability

The data and materials that used in this study and support the findings are available at Mendeley Data (doi: 10.17632/2svx5p9kyz.1 & doi: 10.17632/5bz5253jb4.1) and from the first and corresponding author, Ostapchuk Aleksey, upon reasonable request.
